# Effect of appropriate dose, spectrum, and timing of antibiotics on 28-day mortality in patients with sepsis in the emergency department

**DOI:** 10.1186/s12245-022-00416-6

**Published:** 2022-03-24

**Authors:** Pitsucha Sanguanwit, Chonpisit Monthonn, Thidathit Prachanukool, Karn Suttapanit

**Affiliations:** Department of Emergency Medicine, Faculty of Medicine Ramathibodi Hospital, Mahidol University, Bangkok, 10400 Thailand

**Keywords:** Appropriate antibiotics, Timing, Dose, Spectrum, Sepsis, 28-day mortality, Emergency department

## Abstract

**Background:**

Appropriate antibiotics prescribing is key to treatment and to preventing mortality in patients with sepsis. The aim of this study was to determine the effect of the appropriate timing, spectrum, and dose of antibiotics on 28-day mortality in patients with sepsis.

**Methods:**

We performed a retrospective cohort observational study. We enrolled patients with sepsis in the emergency department of a tertiary care hospital between 1 March and 31 July 2019. Patients were coded into an appropriate antibiotics group (time, spectrum, dose) and an inappropriate antibiotics group. We collected information of patient characteristics, comorbidities, vital signs, laboratory test results, and initial treatment. We followed patient outcomes, 28-day mortality, hospital deaths, 28-day ventilator-free days, and 28-day hospital-free days.

**Results:**

A total of 593 patients were enrolled, with 323 (54.46%) in the appropriate antibiotics group. We used multivariate logistic analyses to assess factors for mortality. Primary outcomes of appropriate antibiotics (administration within 60 min of triage, appropriate spectrum and dose) did not affect 28-day mortality (adjusted odds ratio [OR], 0.57; 95% confidence interval [CI] 0.22–1.144; *P*=0.23). Subgroup analysis showed that appropriate spectrum alone influenced 28-day mortality (adjusted OR, 0.38; 95% CI, 0.15–0.99; *P*=0.047). Appropriate antibiotics was not associated with in-hospital mortality (adjusted OR, 0.62; 95% CI, 0.29–1.30; *P*=0.21).

**Conclusion:**

Appropriate antibiotics included timing less than 60 min, spectrum and the dose was not significantly affected in 28-day mortality in emergency sepsis patients.

**Trial registration:**

The trial was retrospectively registered in the Thai Clinical Trial Registry, identification number TCTR20211216003.

**Supplementary Information:**

The online version contains supplementary material available at 10.1186/s12245-022-00416-6.

## Introduction

Sepsis involves life-threatening organ dysfunction caused by a dysregulated host response to infection [1]. Sepsis may affect multiple organ systems, such as in respiratory, urinary tract, and primary septicemia, and its mortality rate is high without proper treatment [2]. The Ministry of Public Health of Thailand reported a high mortality rate owing to sepsis of 32% in 2018 [3].

The Third International Consensus Definitions for Sepsis and Septic Shock (SEPSIS-3) advocates using the Sequential Organ Failure Assessment (SOFA) score to evaluate sepsis. The quick SOFA (qSOFA) score was developed as a better predictor of mortality in outside the intensive care unit (ICU) and emergency department (ED) settings than the SOFA score and SIRS [1]. Sepsis involves a multi-system physiologic response with an increased respiratory rate (RR) or tachypnea (RR > 20 breaths per minute), hypotension (mean arterial blood pressure less than 60 mmHg), and altered level of consciousness (decreased Glasgow Coma Score). Respiratory rate, hypotension, and altered level of consciousness were included in calculating the qSOFA score [4], related to the mortality rate [5]. Therefore, early recognition of sepsis and effective management in the ED during the early stages can improve patient outcomes [6].

In the Surviving Sepsis Campaign from the International Guideline for Management of Sepsis and Septic Shock: 2018 Update, 1-Hour Bundle Sepsis Campaign [7], standard treatment in sepsis patients involves early administration of broad-spectrum antibiotics. The guidelines are important to improving patient outcomes, survival rates, and reducing hospital stay length.

Current data indicate that each hour of delay in antibiotics administration for patients with sepsis and septic shock increases the mortality rate [8, 9], length of hospital stay [10], and the risk of acute kidney injury [11] and acute lung injury [12]. The Surviving Sepsis Campaign: International Guidelines for Management of Sepsis and Septic Shock: 2016 [13] recommend administering early antibiotics within the first hour from time zero. However, in 2020, the Infectious Diseases Society of America (IDSA) expressed concern about the definition of time zero and a tentative diagnosis of sepsis [14]. IDSA revised the National Severe Sepsis and Septic Shock Early Management Bundle (SEP-1) Sepsis Quality Measure in 2015, recommended broad-spectrum antibiotics in septic shock, and administered empiric antibiotics appropriately as soon as possible in sepsis because a variety of diseases can mimic sepsis. Recent data show that antibiotics administration 1 h from sepsis triage has little association with 28-day mortality [15, 16] but increases mortality in delayed antibiotics administration [17, 18].

Recent data show much information on the effect of appropriate timing of antimicrobial in sepsis patients in the ED, although appropriate spectrum and dose had limited data. Our data could be increased information about spectrum and dose in a tertiary university hospital in Asian, especially in ED that diagnosis and treatments had challenged against timing.

The antibiotics should be selected according to the source of infection, patient immunity, and risk of hospital-acquired infection. Therefore, the first dose of antibiotics should be broad for infection sources, considering pharmacokinetics and pharmacodynamics. Then, physicians can select a single- or multidrug regimen [13].

The appropriate dose of antibiotics is essential in sepsis management. The efficacy of many drugs depends on peak blood level (dose-dependent) and minimum inhibitory concentration (time-dependent). Initiating a loading dose as a guideline and the appropriate therapeutic blood level will be reached quickly. The next dose depends on the volume distribution of the drug or the level.

This study aimed to evaluate the effect of the appropriate dose, spectrum, and timing of antibiotics administration on outcomes of 28-day mortality, in-hospital mortality, 28-day ventilator-free days, and 28-day hospital-free days among patients with sepsis in the ED.

## Methods

### Study design and patient cohort setting

We performed a retrospective cohort observational study. We enrolled patients diagnosed with sepsis who visited the ED of Ramathibodi Hospital, a supra-tertiary university hospital in Bangkok, Thailand, between March 1 and July 31, 2019. The diagnosis of sepsis was made according to the International Classification of Diseases, Tenth Revision (ICD 10), by the attending physician or based on the results of blood, body fluid, or specimen culture. Informed consent was waived as the data were retrospectively collected and were anonymous. This study was approved by The Committee on Human Rights Related to Research, Faculty of Medicine Ramathibodi Hospital, Mahidol University (IRB COA. MURA2019/648 Date July 25, 2019).

### Study participants

We screened all Ramathibodi ED sepsis protocol records during the study period for eligibility. Patients who fulfilled all of the following criteria were included in the analysis: (1) aged > 15 years; (2) diagnosed with sepsis according to the ICD-10 or by an attending physician or according to the results of blood culture, body fluid culture, or specimen culture; and (3) patients in the ED in whom the Ramathibodi sepsis protocol had been followed (Supplement 1 and 2). The exclusion criteria were (1) patients who had received oral antibiotics within the past 7-day; (2) patients with cardiac arrest before arrival in the ED or with a do-not-resuscitate order signed on the day of arrival in the ED; (3) patients with a definite diagnosis unrelated to sepsis; 4) patients who were transferred out of the ED within 24 h; (5) patients referred in from other hospitals; and (6) patients with missing data in the Ramathibodi Hospital database and emergency medical record.

### Treatment group

This study was in the ED of a supra-tertiary university hospital in Bangkok, Thailand. The included patients were coded into one of two groups according to antibiotic treatment. The appropriate antibiotics group was defined as patients with all of the following: (1) appropriate time of antibiotics administration (measured as the receipt of the first dose within 60 min from the time of triage); (2) appropriate spectrum (defined in a review of the results of hemoculture or specimen culture, or an antibiotics spectrum covering the suspected source pathogen; patients with any infection source or negative blood culture results were assumed to have received appropriate antibiotics if received antibiotics according to the type of pathogen, type of the previous antibiotic, and history of the previous admission or given antibiotics [19]); and (3) appropriate dose (the first dose used the normal glomerular filtration rate (GFR) dose according to the Stanford Health Care Antimicrobial Dosing Reference Guide) [20]. The inappropriate antibiotics group was defined as patients with any one of the following: (1) inappropriate time (not meeting the above definition), (2) inappropriate spectrum (not meeting the above definition), and (3) inappropriate dose (not meeting the above definition) [20]. Both groups of patients received standard care in the ED according to the Ramathibodi emergency sepsis protocol.

### Study endpoints

The primary endpoint was the proportion of 28-day mortality in the appropriate antibiotics group; 28-day mortality was defined as all-cause mortality within 28-day of disease. The secondary endpoints were hospital mortality (death status at hospital discharge), 28-day ventilator-free days (days alive without use of a ventilator at 28-day from sepsis diagnosis), and 28-day hospital-free days (days alive without hospitalization at 28-day).

### Data collection

**Fig. 1:**
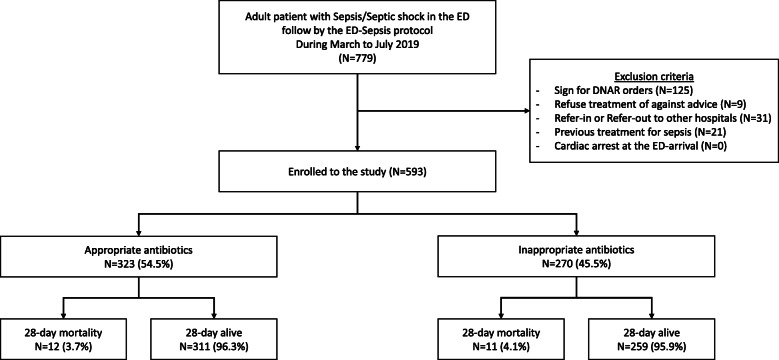
Study flow chart

We collected information on patient characteristics, including age, sex, underlying conditions, vital signs at triage (systolic blood pressure [BP], diastolic BP, heart rate, body temperature, RR, and oxygen saturation [SpO_2_]). In addition, we calculated qSOFA and SOFA scores. Initial laboratory findings (white blood cell count, platelet count, serum creatinine, initial serum lactate) and volume of fluids given in the first hour were collected. Patients were given antibiotics according to the 1-h bundle sepsis protocol of Ramathibodi Hospital. Details of antibiotics, time interval to the first dose, the dose of first antibiotics, and spectrum of antibiotics were recorded and analyzed for the appropriateness of each parameter. In addition, information regarding the source of infection, source pathogen of positive cultures, the pathogen of positive hemoculture, appropriate time of antibiotics administration, an appropriate spectrum of antibiotics, and appropriate dose of antibiotics was documented. The protocol is illustrated in Fig. 1.

### Sample size and statistical analyses

The sample size was calculated using the proportion of patients with sepsis in the ED who died after using the Bundle Sepsis Protocol between September 2015 and August 2016 was 5.65%. The sample size in the unexposed group was calculated using the mortality rate before using the 3-Hour Bundle Sepsis Protocol (Timing Antibiotic within 60 min), between January and December 2014 was 14.2%.

The sample size was calculated using the N4 software application for cohort studies, in which *P* (outcome/exposure) = *p*1 = 0.0565, *P* (outcome/unexposed) = *p*2 = 0.142, ratio 1:1, alpha (*α*) = 0.05, and beta (*β*) = 0.20. The sample size using a continuity correction was unexposed = 211 and exposed = 211.

Descriptive statistics were calculated for all clinical characteristics and relevant variables; continuous variable data are presented as mean (standard deviation; SD) with a normal distribution or median in non-parametric tests and using an independent *t*-test or Mann–Whitney *U* test. Categorical data are presented as percentages using a chi-square test or Fisher’s exact test, as appropriate. Primary and secondary analyses of 28-day and hospital mortality were compared using multivariable logistic regression for binary outcomes. We compared 28-day ventilator-free and 28-day hospital-free days using a log transformation to correct the outcomes to the normal distribution and analyze them with a multivariable linear regression model. All tests were two-sided and values were considered to be statistically significant with a P-value less than 0.05. We performed all data analysis using Stata version 16 (StataCorp LLC, College Station, TX, USA).

## Results

In total, 779 patients were screened from March to July 2019, among whom 186 patients failed to meet the inclusion criteria. A total of 593 participants met the eligibility criteria. In total, 323 patients were included in the appropriate antibiotics group (all received the appropriate antibiotics dose, spectrum, and administration time within 60 min) and 270 patients were included in the inappropriate antibiotics (had any one of the following: inappropriate antibiotics dose (*n*=2; 0.34%), inappropriate antibiotics spectrum (*n*=104; 17.54%), or administration time longer than 60 min (*n*=198; 33.39%)). The primary outcome of 28-day mortality was 3.7% (12/323) in the appropriate antibiotics group and 4.1% (11/270) in the inappropriate antibiotics group (Fig. 1).

Baseline characteristics are presented in Table 1. Overall, patients’ age, gender, underlying diseases, and vital signs at triage were generally similar between the two groups, except a higher proportion of patients in the appropriate antibiotics group had heart disease. Additionally, mean RR was increased in the appropriate antibiotics group (24.78±5.1 vs. 23.07±4.38 bpm; *P*< 0.001) and mean oxygen saturation was decreased (94.38%±7.5% vs. 97.07%±2.98%; *P*< 0.001]. Laboratory findings including white blood cells, platelet, serum creatinine, initial serum lactate, and initial SOFA score were similar between the group, but qSOFA was statistically significant in the appropriate antibiotics group (median 1 interquartile range [IQR] 1,1 vs. 1 IQR 0,1), as illustrated in Table 1.

**Table 1:** Characteristics of appropriate antibiotics group (time < 60 min, spectrum, dose) and inappropriate antibiotics group

Characteristics	Appropriate antibiotics *N*=323	Inappropriate antibiotics *N*=270	*P* value
Age (years), mean ± SD	70.7±16.91	67±18.02	0.62
Gender			
Male, *n* (%)	151 (46.7%)	115 (42.6%)	0.31
Underlying conditions, *n* (%)			
Hypertension	183 (56.7%)	140 (51.9%)	0.24
Diabetes mellitus	115 (35.6%)	100 (37%)	0.72
Chronic kidney disease	86 (26.6%)	69 (25.6%)	0.77
Heart disease	88 (27.2%)	50 (18.5%)	0.01
Liver disease	35 (10.8%)	25 (9.3%)	0.53
Immunocompromised	111 (34.4%)	101 (37.4%)	0.44
Vital signs at triage, mean ± SD			
Systolic BP (mmHg)	133.18±32.25	129.26±29.25	0.125
Diastolic BP (mmHg)	71.84±15.58	70.86±13.77	0.42
Heart rate (bpm)	108.05±23.21	107.38±20	0.72
Body temperature (°C)	38.28±1.1	38.32±0.99	0.57
Respiratory rate (bpm)	24.78±5.1	23.07±4.38	< 0.001
Oxygen saturation (%)	94.38±7.5	97.07±2.98	< 0.001
Laboratory results, mean ± SD			
White blood cell count^a^ (× 10^3^)	10.0 (6.7, 13.7)	9.95 (6.8, 13.2)	0.95
Platelet count^a^ (×10^3^)	207.0 (147, 280)	211.5 (154, 284)	0.90
Serum creatinine^a^	0.98 (0.72, 1.48)	0.93 (0.68, 1.39)	0.50
Initial serum lactate (mmol/L), mean ± SD	2.54±1.61	2.41±1.61	0.36
qSOFA score^a^	1 (1,1)	1 (0,1)	< 0.001
SOFA score^a^	1 (0,3)	1 (0,3)	0.24
Severity			
Sepsis, *n* (%)	295 (91.3%)	245 (90.7%)	0.80
Septic shock, *n* (%)	28 (8.7%)	25 (9.3%)	
Time triage to antibiotics^a^ (minutes)	39 (30, 50)	73 (55, 101)	< 0.001
Fluids in first hour^a^ (mL)	80 (80, 400)	100 (80, 400)	< 0.001

^a^Median (interquartile range); rank-sum test

*SD* standard deviation, *BP* blood pressure, *qSOFA* quick Sequential Organ Failure Assessment score

The factors chosen for multivariable logistic regression analysis of primary outcome (28-day mortality) and secondary outcome were qSOFA, RR, and SpO_2_. After the adjusting, the primary outcome in the appropriate antibiotics group showed no effect on 28-day mortality and in-hospital mortality (adjusted odds ratio [OR], 0.57; 95% confidence interval [CI], 0.22–1.144); adjusted OR, 0.62; 95% CI, 0.29–1.30, respectively). However, we observed an effect according to the mean difference in 28-day ventilator-free days and 28-day hospital-free days (adjusted coefficient 1.06 [0.17, 1.94], *P*=0.02), (adjusted coefficient 1.55 [0.16, 2.93], *P*=0.03), shown in Table 2.

**Table 2:** Comparison of primary and secondary outcomes between appropriate antibiotics (time, spectrum, dose) and inappropriate antibiotics groups

Outcomes	Appropriate antibiotics ***N***=323	Inappropriate antibiotics ***N***=270	Adjusted odds ratio^**a**^ Adjusted coefficient^**b**^: Appropriate antibiotics	***P*** value
**Primary outcomes**				
28-day mortality, *n* (%)	12 (3.7%)	11 (4.1%)	0.57 (0.22, 1.44)^a^	0.23
In-hospital mortality, *n* (%)	19 (5.9%)	17 (6.3%)	0.62 (0.29, 1.30)^a^	0.21
28-day ventilator- free days, mean ± SD	26.35±5.13	26.13±6.06	0.01 (− 0.03, 0.05)^b^	0.57
28-day hospital-free days, mean ± SD	20.70±8.30	20.27±9.17	0.03 (− 0.05, 0.11)^b^	0.49

^a^Adjusted odds ratio (95% confidence interval): qSOFA, respiratory rate, oxygen saturation

^b^Apply log transformation for the fitting model to normal distribution before Adjusted coefficient (mean difference): qSOFA, respiratory rate, oxygen saturation. After we did a log transform, 16 data of 28-day ventilator-free day and 57 data of 28-day hospital free day were missing because log [0] transformed to infinities. We had completed case analysis as 577 patients for 28-day ventilator-free day and 536 patients 28-day hospital free day outcomes

*SD* standard deviation, *qSOFA* quick Sequential Organ Failure Assessment

In subgroup analysis, appropriate spectrum alone affected 28-day mortality and in-hospital mortality (adjusted OR, 0.38; 95% CI, 0.15–0.99; *P*=0.047 and adjusted OR, 0.28; 95% CI, 0.13–0.61; *P*=0.001, respectively); this was not associated with the mean difference 28-day ventilator-free day and mean difference in 28-day hospital-free day (adjusted coefficient, 0.06; 95% CI, 0.01–0.11; *P*=0.01) and trend to increase 28-day hospital-free days (adjusted coefficient, 0.10; 95% CI, 0.00–0.21; *P*=0.06, respectively), as shown in Table 3. However, an appropriate time to antibiotics alone did not influence 28-day mortality (adjusted OR, 1.23; 95% CI, 0.32–4.75; *P*=0.76) after adjustment for appropriate spectrum, RR, SpO_2_, and qSOFA.

**Table 3:** Comparison of outcomes between spectrum in appropriate antibiotics and inappropriate spectrum antibiotics groups

	Appropriate antibiotics spectrum, ***N***=489	Inappropriate antibiotics spectrum, ***N***=104	Adjusted odds ratio^**a**^ Adjusted coefficient^**b**^: Appropriate antibiotics	***P*** value
28-day mortality, *n* (%)	14 (2.9%)	9 (8.78%)	0.38 (0.15, 0.99) ^a^	0.047
In-hospital mortality, *n* (%)	21 (4.3%)	15 (14.4%)	0.28 (0.13, 0.61) ^a^	0.001
28-day ventilator- free days, mean ± SD	26.77±4.46	23.75±8.73	0.06** (0.01, 0.11) ^**b**^	0.01
28-day hospital-free days, mean ± SD	21.27±8.08	16.90±10.48	0.10** (− 0.00, 0.21) ^**b**^	0.06

^a^Adjusted odds ratio (95% confidence interval): qSOFA, respiratory rate, oxygen saturation

^b^Apply log transformation for fitting model to normal distribution before adjusted coefficient (mean difference): qSOFA, respiratory rate, oxygen saturation. After we did a log transform, 16 data of 28-day ventilator free day and 57 data of 28-day hospital free day were missing because log [0] transformed to infinities. We had completed case analysis as 577 patients for 28-day ventilator free day and 536 patients 28-day hospital free day outcomes

*SD* standard deviation, *qSOFA* quick Sequential Organ Failure Assessment

In subgroup analysis, patients who had SOFA score ≥ 2 showed in Table 4. The appropriate antibiotics group showed no effect on 28-day mortality, in-hospital mortality, 28-day ventilator-free day, and 28-day hospital-free days.

**Table 4:** Comparison of primary and secondary outcomes between appropriate antibiotics and inappropriate antibiotics groups in subgroup patients who had SOFA ≥ 2

Outcomes	Appropriate antibiotics ***N***=155	Inappropriate antibiotics ***N***=115	Adjusted odds ratio^**a**^ Adjusted coefficient^**b**^: Appropriate antibiotics	***P*** value
**Primary outcomes**				
28-day mortality, *n* (%)	9 (5.81%)	9 (7.83%)	0.60 (0.19, 1.77) ^a^	0.35
In-hospital mortality, *n* (%)	59 (9.68%)	14 (12.17%)	0.68 (0.29, 1.60) ^a^	0.38
28-day ventilator- free days, mean ± SD	25.35±6.70	25.36±7.04	− 0.01 (− 0.09, 0.06) ^b^	0.71
28-day hospital-free days, mean ± SD	18.96±8.94	17.95±9.84	0.08 (− 0.05, 0.22) ^b^	0.21

^a^Adjusted odds ratio (95% confidence interval): qSOFA, respiratory rate, oxygen saturation

^b^Apply log transformation for the fitting model to normal distribution before Adjusted coefficient (mean difference): qSOFA, respiratory rate, oxygen saturation. After we did a log transform, 12 data of 28-day ventilator-free day and 36 data of 28-day hospital free day were missing because log [0] transformed to infinities. We had completed case analysis as 258 patients for 28-day ventilator-free day and 234 patients 28-day hospital-free day outcomes

*SD* standard deviation, *qSOFA* quick Sequential Organ Failure Assessment, *SOFA* Sequential Organ Failure Assessment

Characteristics of infection source among survivors and non-survivors at 28-day were shown in Table 5. Source of infection and source positive culture were generally similar among survivors and non-survivors at 28 days; most sources were pneumonia, urinary tract infection, and gastrointestinal infection. The pathogen in positive culture was not statistically significant, the most common pathogen was *Escherichia coli* and *E. coli* (ESBL), and the culture was no growth around 61%. Infection source sensitivity to first dose antibiotic, appropriate time to antibiotics (within 60 min), and appropriate spectrum of antibiotics were statistically significant in the survival group. Hemoculture sensitivity to the first antibiotic, an appropriate dose of antibiotics, and appropriate antibiotics (time < 60 min + spectrum + dose) did not differ between survivors and non-survivors groups.

**Table 5:** Characteristics of infection source among survivors and non-survivors at 28 days

Characteristics, ***n*** (%)	28-day survival, ***N***=570	28-day mortality, ***N***=23	***P*** value
**Source**			0.64
Pneumonia	195 (34.2%)	9 (31.9%)	
Urinary tract	212 (37.2%)	5 (21.7%)	
Gastrointestinal	55 (9.6%)	5 (21.7%)	
Soft tissue, bone, and joints	31 (5.4%)	2 (8.7%)	
Septicemia/catheter-relate/FN	58 (10.2%)	2 (8.7%)	
Hepatobiliary	10 (1.8%)	0 (0%)	
CNS	2 (0.4%)	0 (0%)	
Unknown	6 (1.1%)	0 (0%)	
Source, positive culture	237(41.6%)	12 (52.2%)	0.31
**Pathogen in positive culture**			0.53
*Escherichia coli*	45 (7.9%)	3 (13%)	
*E. coli* (ESBL)	58 (10.2%)	2 (8.7%)	
*Klebsiella*	21 (3.7%)	0 (0%)	
*Klebsiella* (ESBL)	9 (1.36%)	2 (8.7%)	
*Pseudomonas aeruginosa*	22 (3.9%)	0 (0%)	
*Staphylococcus* spp.	12 (2.1%)	1 (4.3%)	
*Streptococcus* spp.	7 (1.2%)	0 (0%)	
Other	46 (8.1%)	5 (21.7%)	
No growth	350 (61.4%)	10 (4.5%)	
Infection source sensitivity to first dose antibiotic	496 (87%)	15 (65.2%)	< 0.01
Hemoculture positive	85 (14.9%)	4 (17.4%)	0.74
**Pathogen in positive hemoculture**			0.25
*Escherichia coli*	16 (2.8%)	0 (0%)	
*E. coli* (ESBL)	16 (2.8%)	1 (4.3%)	
*Klebsiella*	3 (0.5%)	0 (0%)	
*Klebsiella* (ESBL)	1 (0.2%)	0 (0%)	
*Pseudomonas aeruginosa*	6 (1.1%)	0 (0%)	
*Staphylococcus* spp.	22 (3.9%)	0 (0%)	
*Streptococcus* spp.	4 (0.7%)	0 (0%)	
Other	15 (2.6%)	3 (13%)	
No growth	487 (85.4%)	19 (82.6%)	
Hemoculture sensitivity to first antibiotic	541 (95.1%)	20 (87%)	0.09
Appropriate time to antibiotics (within 60 min)	375 (65.8%)	20 (87.0%)	0.04
Appropriate spectrum of antibiotics	475 (83.3%)	14 (60.9%)	0.005
Appropriate dose of antibiotics	568 (99.6%)	23 (100%)	1.00 ^a^
Appropriate antibiotics (time <60 min + spectrum + dose)	311 (54.6%)	12 (52.5%)	0.82

^a^Fisher’s exact test

## Discussion

The Surviving Sepsis campaign is one of the most important tools to treat sepsis and decrease mortality. Hemoculture, lactate level, and adequate fluid resuscitation are critical in sepsis treatment, as well as the use of antibiotics [13]. The latest 3-h bundle to 1-h bundle sepsis protocol recommends that earlier antibiotics administration predict better outcomes in patients with sepsis [7, 13]. Three areas of focus in current research in patients with sepsis have indicated that patients should receive the appropriate drug at the right time and dose. Previous studies showed better outcomes focusing only on the right time when they got antibiotics earlier than 1 h [21].

The Surviving Sepsis campaign 2021 recommend administering antimicrobials immediately, ideally within 1 h for adults with definite sepsis or probable and shock patient with possible sepsis. In possible sepsis patients without shock, they suggested an immediate investigation if concern for infection persists and antibiotics administration within 3 h [22]. The sepsis treatment bundle was a challenge for the physicians. If we administer too late antibiotics in probable sepsis, that will affect mortality. On the other hand, if we administer early antibiotics in non-shock possible sepsis, we may overuse antibiotics in those who did not finally diagnose sepsis.

In the primary outcome, the data showed that appropriate antibiotics (appropriate timing within 1 h, spectrum, and dose) did not affect 28-day mortality (adjusted OR, 0.57; 95% CI, 0.22–1.144) or in-hospital mortality. For mortality and secondary outcome, the adjusted covariates included respiratory rate SpO_2_ and qSOFA; we did not use fluid received in the 1-h bundle due to intravenous fluid received (80–100 ml).

Our research was the earlier study of the effect of antibiotics composing not only time to administration but also spectrum and dose in patients with sepsis that used the 1-h bundle for treatment in the ED.

Appropriate rational use must be composite of good quality in the right drug, dose, and time. In subgroup analysis, the appropriate only time to antibiotics (within 60 min) was not associated with 28-day mortality (adjusted OR, 1.23; 95% CI, 0.32–4.75; *P*=0.76) after adjusting for appropriate spectrum, RR, SpO_2_, and qSOFA.

Berrevoets et al. [2] reported the appropriateness of antibiotics using seven quality indicators (in terms of the intravenous route in sepsis, antibiotics within 3 h, prior hemoculture and specimen culture, antibiotics plan, prescribed according to guidelines, and adjusted dose) in the ED. Those authors found that appropriate antibiotic use in seven indicators was associated with reduced in-hospital mortality. They did not only focus on time, prescribed according to guidelines, dose, route, and plan. Our study used within 60 min to indicate appropriate timing, following the Surviving Sepsis Campaign in 2018 [7], focusing the right spectrum followed Stanford Antimicrobial Safety and Sustainability Program [19] and Ramathibodi Antibiotic Guide for Sepsis and Septic Shock, the right dose followed Stanford Health Care Antimicrobial Dosing Reference Guide [20].

Fibin et al. [23] reported no difference for in-hospital mortality before and after quality improvement in sepsis care, including administration of antibiotics within the first hour after triage. That result implies that the appropriate time to administer antibiotics in our population who were less severe with sepsis or the possible sepsis patients can be between “as soon as possible” to within 3 h. That may be compatible with recent data from The Surviving Sepsis campaign 2021 in the timing of antibiotics. Our study population has less severe sepsis due to screening from patients who present clinically possible sepsis in the ED and mostly community acquire. If we cut point inappropriate time more than 3 h in the less severe group, it may show a different outcome; however, the appropriate spectrum is important in 28-day mortality.

In the subgroup who had SOFA score ≥ 2 (Table 4), the appropriate antibiotics (appropriate timing within 1 h, spectrum, and dose) did not affect 28-day mortality or in-hospital mortality. However, we need more sample size to analyze mortality outcomes in severe or septic shock groups.

Only the appropriate spectrum of antibiotics subgroup was associated with decreased 28-day mortality (adjusted OR, 0.38; 95% CI, 0.15–0.99; *P*=0.047) and decrease in-hospital mortality (adjusted OR, 0.28; 95% CI, 0.13–0.61; *P*=0.001) (Table 3). Subgroup analysis for appropriate antibiotics dose was limited due to the small sample size for non-appropriate doses. From our data, that seems to be the right spectrum was more affected to 28-day mortality than the timing within 60 min.

We analyzed spectrum appropriateness according to two criteria. First, the source of infection developed sepsis and positive cultures received antibiotics which is proper antibiotics. In the source, there are suspected negative cultures, appropriate antibiotic-related source, and underlying disease, prior antibiotics, risk of hospital-acquired infection, and prior hospital admission. The appropriate spectrum of antibiotics seems to affect mortality, so updating the local antibiogram and following the antibiotic guideline protocol may improve the appropriate prescribing and decrease mortality.

The other secondary outcome, appropriate antibiotics (timing within 1 h, appropriate spectrum and dose), was not associated with an increased mean difference in 28-day ventilator-free and 28-day hospital-free days. In the subgroup, the appropriate spectrum of antibiotics seems to be associated with a 28-day ventilator and hospital-free day. In terms of clinical outcomes, this seems to be a small effect. However, in situations with overcrowding in the ED and a lack of available inpatient beds, appropriate antibiotics could affect the flow of treatment and the resuscitation process in the ED.

Based on our study findings, we can conclude that appropriate antibiotics timing within 1 h. The spectrum and dose do not affect 28-day mortality. It may be the inconclusive timing of antibiotics, and our data were in supra-tertiary care in urban areas. However, this result may support the recommendation of surviving sepsis campaign guideline; we advocated that the timing of administering antimicrobials must be categorized by probability and severity of sepsis. If the patients were high probability or high severity of sepsis, they should receive antimicrobials within 1 h. If the patients were low probability and low severity of sepsis, they should receive antimicrobials within 3 h; moreover, we recommend providing antibiotics with the appropriate spectrum as local antibiogram, following international or local guidelines, the appropriate dose in patients with sepsis in the ED.

## Limitations

There are some limitations to this study. Firstly, our data were from a single, supra-tertiary care center at a medical school, in which patients may have received appropriate antibiotics more frequently than in other similar-sized hospitals or those in rural areas. Secondly, it may have some discrepancies in the diagnosis of sepsis due to the variety of sepsis scores for screening, physician diagnosis, and the ICD-10 code of sepsis, leading to selection or misclassification bias; we had limited in assessing an inter-observer variability. However, the medical record was audited by an internal auditor in Ramathibodi Hospital, which uses medical record audit guideline 2021 by the Healthcare Accreditation Institute of Thailand (Public Organization) [24]. However, no international standard recommends a “gold standard” test to diagnose sepsis.

Thirdly, our population data had less severe sepsis and low mortality outcome; it seems to be under-power to detect primary or secondary outcomes. It seems that our study population was less severe, although mortality was similar to the Self WH et al. [25] non-critically ill sepsis patients from ED.

Fourthly, our research did not investigate antibiotics timing related to triage level; patients who were triaged as more critical may have received earlier antibiotics. Finally, we used vital signs at triage adjusted for clinical outcome.

Fifthly, in the recognition of the appropriated spectrum, we reviewed for sensing antibiotics and included any broad-spectrum antibiotics that covered positive bacterial cultures, and there was no growth regarding antibiotics spectrum. Clinical suspicious cause of antibiotics with no culture result can be an improper corrected source or misdiagnosis included in proper antibiotics.

Sixthly, recognizing appropriate dose, the first dose of antibiotics is mostly full, as recommended. Therefore, the number of patients who received inappropriate doses is deficient. However, we had limited data regarding the next dose of antibiotics, the duration, administration route, and antibiotic stewardship.

Further studies should be prospective and include a larger sample size for detecting more mortality outcomes. The data of quality of administration of antibiotics in sepsis such as antibiotics timing, prior hemoculture and specimen culture, antibiotics plan, prescribed according to guidelines, and adjusted dose in the ED might be needed.

## Conclusions

Our cohort study conducted among patients with sepsis in the ED revealed that appropriate timing of antibiotics administration within1 h, spectrum, and dose was not associated with 28-day mortality or in-hospital mortality and was related to increased 28-day ventilator-free days and 28-day in-hospital-free days. Only the subgroup appropriate antibiotics spectrum was associated with decreased 28-day mortality, in-hospital death, and increased ventilator-free days and hospital-free at 28 days.

## Supplementary Information

**Additional file 1.** ER Sepsis Protocol.

**Additional file 2.** Sepsis screening tool.

## Data Availability

The datasets analyzed in this study are not publicly available owing to privacy issues but are available from the corresponding author upon reasonable request.

## Abbreviations

CI: Confidence interval
ED: Emergency department
GFR: Glomerular filtration rate
ICU: Intensive care unit
IDSA: Infectious Diseases Society of America
IQR: Interquartile range
OR: Odds ratio
qSOFA: Quick Sequential Organ Failure Assessment
SD: Standard deviation
SOFA: Sequential Organ Failure Assessment
SpO_2_: Oxygen saturation
