# Supplementary material for: Effect of appropriate dose, spectrum, and timing of antibiotics on 28-day mortality in patients with sepsis in the emergency department
Source: Int J Emerg Med. 2022 Mar 24;15:13. doi: 10.1186/s12245-022-00416-6 (PMC8943924; doi:10.1186/s12245-022-00416-6)
Supplement: Supplementary file 1 — Additional file 1. ER Sepsis Protocol. [file 12245_2022_416_MOESM1_ESM.pdf]

Ramathibodi Sepsis Protocol

Version 2020_10

**Hour-1 Sepsis** / Progress	Date/Time	**Hour-1 Sepsis** / Order
**Time zero** = ED Triage =		❑ CBC, E'Lyte, BUN/Cr ❑ POC-glu
Adult REWs ≥4 and suspected infection		❑ H/C x II ❍ LFT, Ca, Mg, PO_4
(Adult REWs table below)		❑ Serum lactate (❑ arterial / ❑ venous)
		❑ Coag ❍ Fibrinogen
		❍ ABG (❍ Room air ❍ O_2 ________________)
		❑ EKG ❑ CXR
**Source**:		❑ U/A, U/C ❍ Sputum G/S, C/S
		❑ Antibiotic STAT (within 1 h)
		❑ IV fluid (if MAP <65, lactate ≥ 4 : initial 30ml/kg)
Initial **Lactate** V…………………A…………………		
(SOFA table below)		❑ Serial lactate (❑ arterial / ❑ venous) at time __________________(if initial lactate>2)
		If MAP <65 : start vasopressor<br>❑ **NorEpinephrine** 4 mg in 5%DW 96 ml IV drip start 5 ml/h (0.05 mcq/kg/min titrate q 5 min)
**SOFA** / baseline (Time ………………)<br>acute change of SOFA score from infection<br>❑ 0-1 : possible discharge if clinically indicated<br>❑ ≥ 2 : Diagnosis of sepsis		❑ **POCUS**

Adult REWs	RR	SpO_2	Temp	SBP	HR	AVPU
3	≤ 10	≤ 84	≤ 33.9	≤ 89	≤ 39	U
2		85-89	34-34.9			P
1		90-92	35-35.9	90-99	40-49	V
0	11-20	≥ 93	36-37.9	100-199	50-99	A
1	21-30		38-38.9		100-109	
2	31-35		≥ 39	≥ 200	110-129	
3	≥ 36				≥ 130	

SOFA	0	1	2	3	4
PaO_2/FiO_2 (mmHg)	≥400	<400	<300	<200 with resp.support	<100 with resp.support
Platelets ($x10^3$)	≥150	<150	<100	<50	<20
T.bilirubin (mg/dL)	<1.2	1.2-1.9	2.0-5.9	6.0-11.9	>12
Cardiovas. (mcg/kg/min at least 1 hr)	MAP≥70	MAP<70	DoPa <5 or DoBu (any dose)	DoPa 5.1-15 or EpiN ≤0.1 or NorE ≤0.1	DoPa >15 or EpiN >0.1 or NorE >0.1
GCS	15	13-14	10-12	6-9	<6
Creatinine (mg/dL)	<1.2	1.2-1.9	2.0-3.4	3.5-4.9	>5
Urine (ml/day)				<500	<200
