# Supplementary material for: Effect of appropriate dose, spectrum, and timing of antibiotics on 28-day mortality in patients with sepsis in the emergency department
Source: Int J Emerg Med. 2022 Mar 24;15:13. doi: 10.1186/s12245-022-00416-6 (PMC8943924; doi:10.1186/s12245-022-00416-6)
Supplement: Supplementary file 2 — Additional file 2. Sepsis screening tool. [file 12245_2022_416_MOESM2_ESM.pdf]

Supplementary 2 Sepsis screening tool

1. quick Sequential organ failure assessment (qSOFA) score ≥ 2

- Hypotension, Systolic BP ≤100 mmHg
- Alteration of consciousness
- Tachypnea ≥22 breath/minute

2. SOFA Score: Sequential organ failure assessment score ≥ 2

	0	1	2	3	4
PaO2/Fio2 Ratio, mmHg	≥400	<400	<300	<200	<100
Platelets, $*10^3$	≥150	<150	<100	<50	<20
Bilirubin, mg/dl	<1.2	1.2-1.9	2.0-5.9	6.0-11.9	>12.0
Cardiovascular (catecholamine Ug/kg/min)	MAP≥70mmHg	MAP<70mmHg	Dopamine<5 or Dobutamine any dose	Dopamine 5.1-15 or epinephrine≤0.1 or norepinephrine <0.1	Dopamine >15 or epinephrine>0.1 or norepinephrine >0.1
Glasgow Coma Scale	15	13-14	10-12	6-9	<6
Creatinine, mg/dl<br>Urine output, ml/day	<1.2	1.2-1.9	1.9-3.4	3.4-5.0<br><500	>5.0<br><200

3. Ramathibodi early warning score (REWs) clinical parameters and rubric scale for each parameter.

REWS						
Score	RR	SpO2	Temperature	SBP	HR	Mental status
3	$\leq$10	$\leq$84	$\leq$33.9	$\leq$89	$\leq$39	
2		85-89	34-34.9			
1		90-92	35-35.9	90-99	40-49	
0	11-20	$\geq$93	36-37.9	100-199	50-99	Alert
1	21-30		38-38.9		100-109	Response to voice
2	31-35		$\geq$39.0	$\geq$200	110-129	Response to pain
3	$\geq$36				$\geq$130	Unresponsive
